# Correction: Transcriptional repression of *reaper* by *Stand still* ensures female germline development in *Drosophila*

**DOI:** 10.1371/journal.pgen.1012116

**Published:** 2026-04-15

**Authors:** The PLOS Genetics Staff

## Notice of Republication

This article was republished on April 2^nd^, 2026, to correct an error in the title: Stand still was erroneously italicized. Please download this article again to view the correct version. The originally published, uncorrected article and the republished, corrected articles are provided here for reference.

## Supporting information

S1 File Originally published, uncorrected article. (PDF)

S2 File Republished, corrected article. (PDF)
