# Supplementary figures and images for: Transcriptional repression of reaper by Stand still ensures female germline development in Drosophila
Source: PLoS Genet. 2026 Mar 5;22(3):e1012041. doi: 10.1371/journal.pgen.1012041 (PMC12962474; doi:10.1371/journal.pgen.1012041)

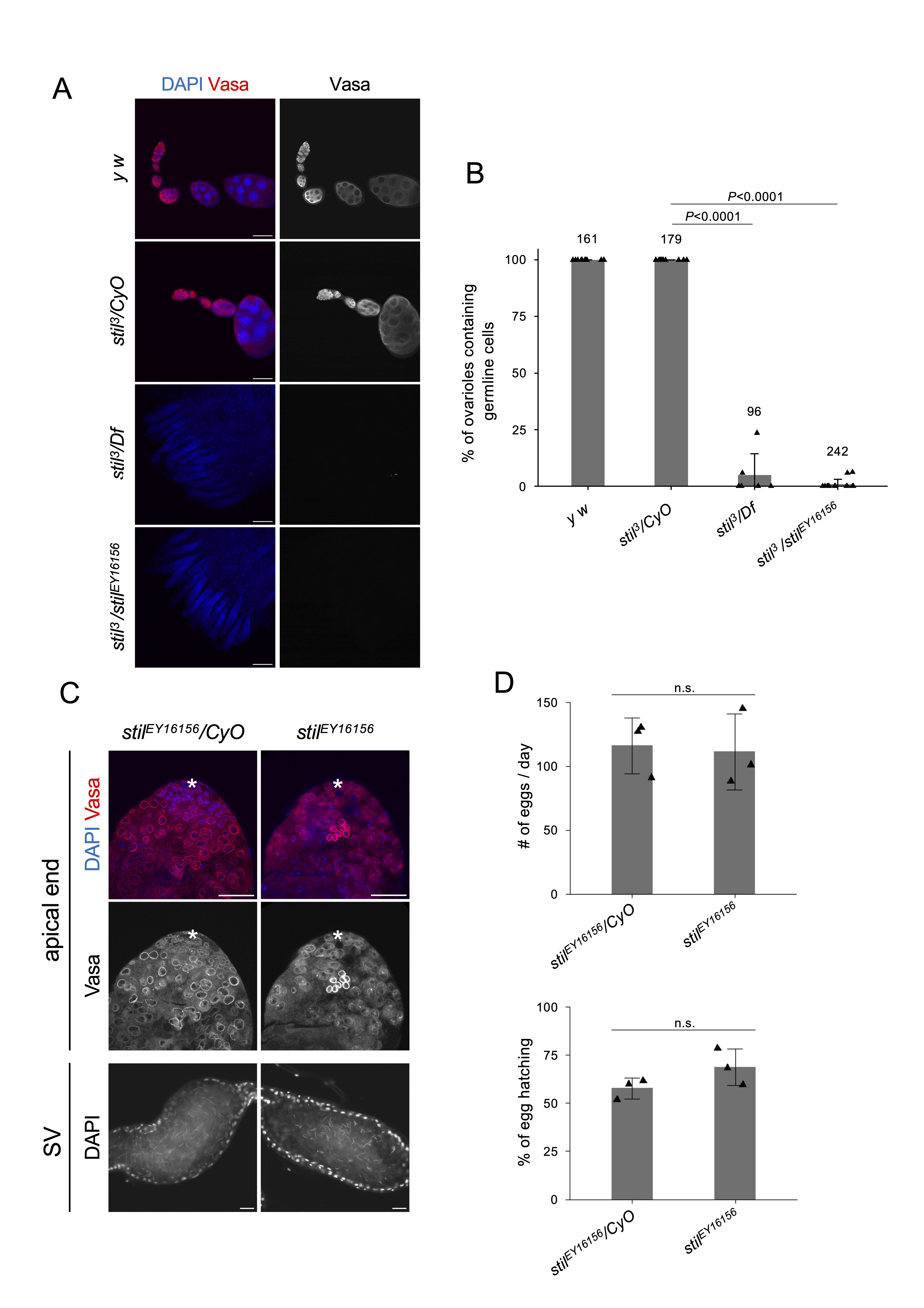

Supplement: S1 Fig — (A) Immunostaining of ovaries from y w, stil3/CyO, stil3/Df, and stil3/stilEY16156 with antibody against Vasa (red) with DAPI (blue). Scale bar: 50 μm. (B) Quantification of the percentage of ovarioles containing germline cells per ovary in 2–3-day-old females. Each dot represents an individual ovary. Genotypes are indicated below the graph and the number of germarium assessed is noted above each bar. Error bars represent standard deviation (s.d.). (C) Immunostaining of the apical end of testes from stilEY16156/CyO and stilEY16156 with anti-Vasa antibody (red) and DAPI (blue) (top panels). Seminal vesicles (SV) harboring sperms are also stained with DAPI (blue) (bottom panels). Asterisk denotes the apex of testis. Scale bar: 50μm (apical end of testes, top) and 20 μm (SV, bottom). (D) The numbers of egg laying and hatching rate. Daily egg laying by three y w females mated with three males of the indicated genotypes are shown (n=3). The number of ovarioles assessed is noted above each bar. Error bar indicates s.d. (JPG) [file pgen.1012041.s001.jpg]

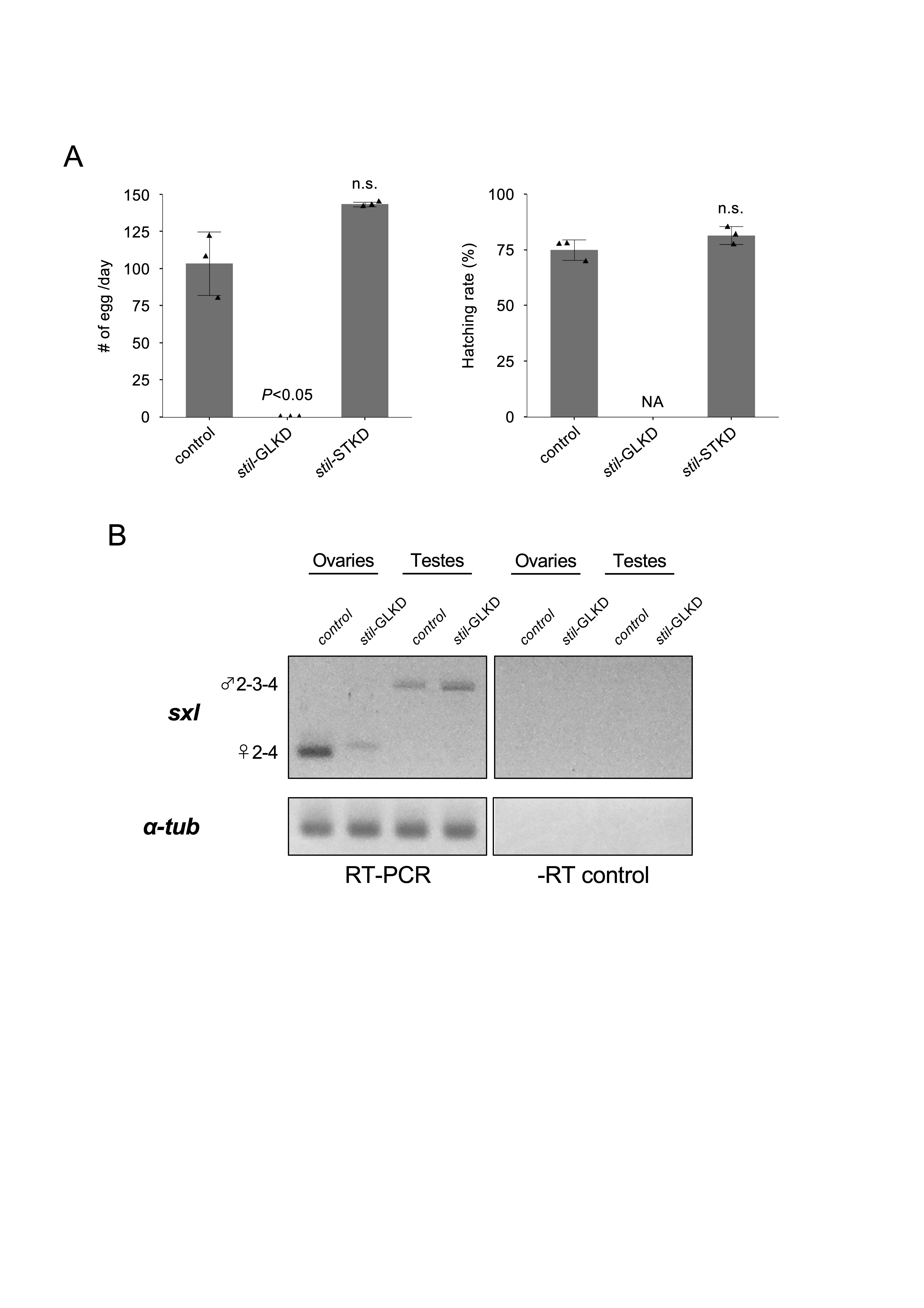

Supplement: S2 Fig — (A) Analysis of egg laying and hatching rates. The number of laid eggs and their hatching rates are measured daily for three females of the indicated genotypes: control, stil-germline knockdown (stil-GLKD) driven by NGT40; NosGal4-VP16, and stil-somatic knockdown (stil-STKD) driven by tj-Gal4, each mated with three y w males (n = 3). Error bars indicate standard deviation (s.d.). (B) RT-PCR analysis of sxl and the control, α-tub transcripts in ovaries and testes from control and stil-GLKD flies, respectively. (JPG) [file pgen.1012041.s002.jpg]

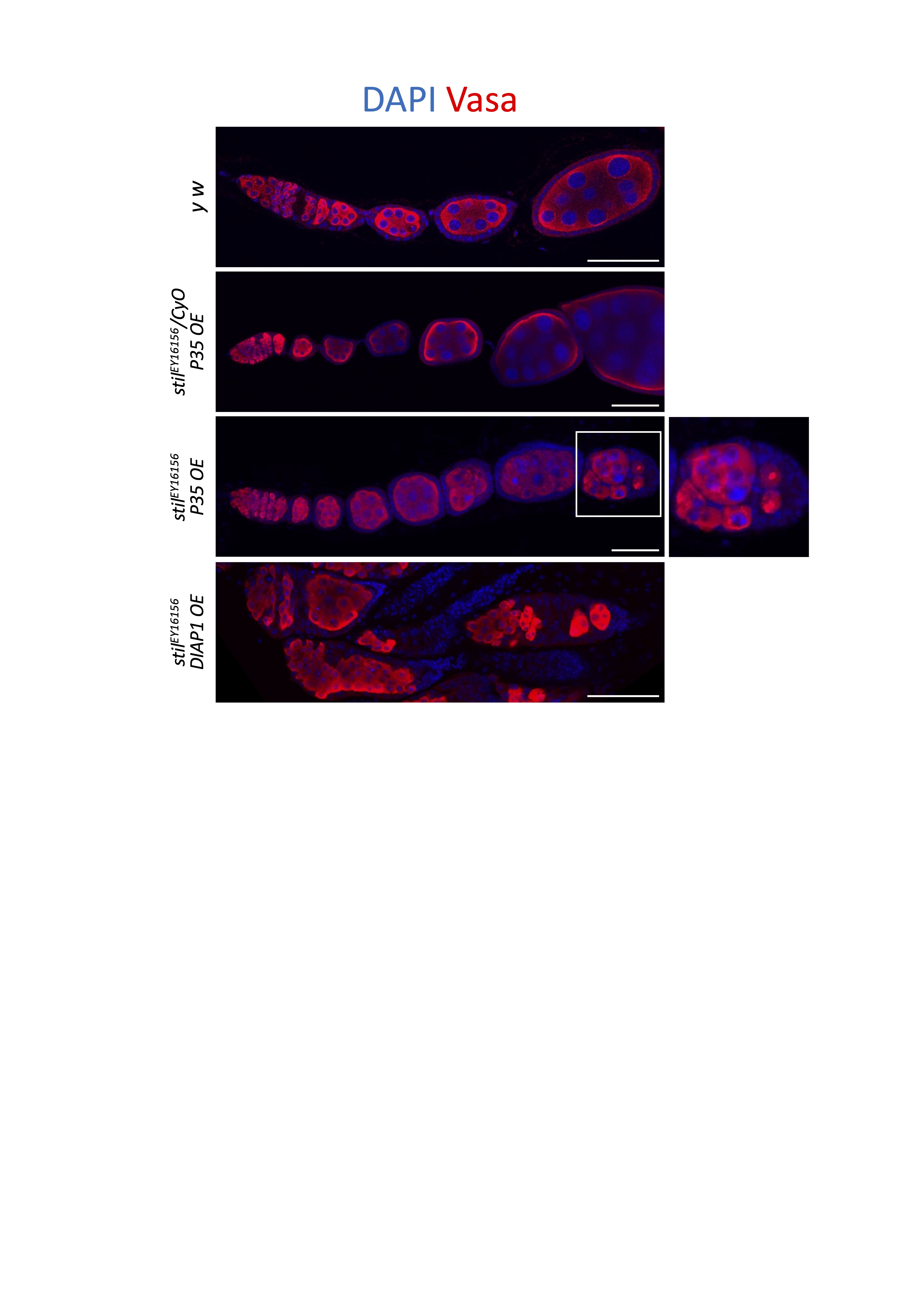

Supplement: S3 Fig — Immunostaining of ovarioles from y w, stilEY16156/CyO; P35 OE (NGT40; NosGal4-VP16 > P35), stilEY16156; P35 OE and stilEY16156; DIAP1 OE (NGT40; NosGal4-VP16 > DIAP1) flies with antibody against Vasa (red) and DAPI (blue). The enlarged image highlights a degenerated egg chamber at the mid-stage of oogenesis in stilEY16156; P35 OE. Scale bar: 50 μm. (JPG) [file pgen.1012041.s003.jpg]

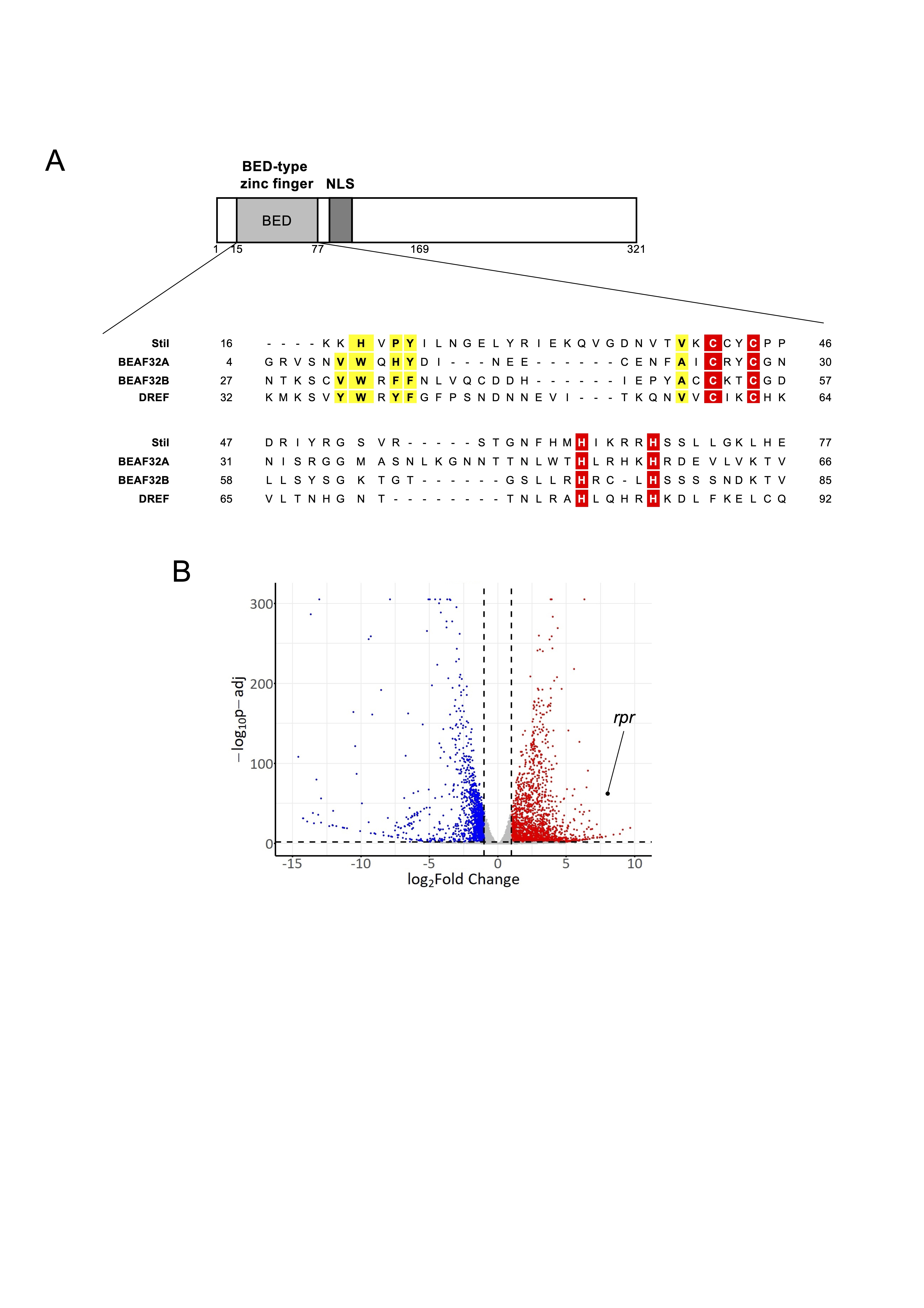

Supplement: S4 Fig — (A) A schematic presentation of Stil protein, highlighting the BED-type zinc finger (BED, green) and the nuclear localization signal (NLS, blue). A multiple sequence alignment of Stil with three other proteins containing BED-type zinc finger motif is shown. The BED-type zinc finger motif consists of two cysteine and histidine residues (red) and aromatic residues (yellow) in the N-terminal region. (B) Transcriptome analysis of germline-rescued stil mutant ovaries expressing P35. Differential expressed genes (DEGs) are obtained with DEseq2. Volcano plot displays the adjusted p-value (p-adj) versus log2-fold change (log2FC) of expression level in stilEY16156; P35 OE compared to that in stilEY16156/CyO; P35 OE. Significantly upregulated genes (log2FC > 1, p-adj < 0.01) and downregulated genes (log2FC <-1, p-adj < 0.01) are highlighted in red and blue, respectively, while non-DEGs are shown in black. (JPG) [file pgen.1012041.s004.jpg]

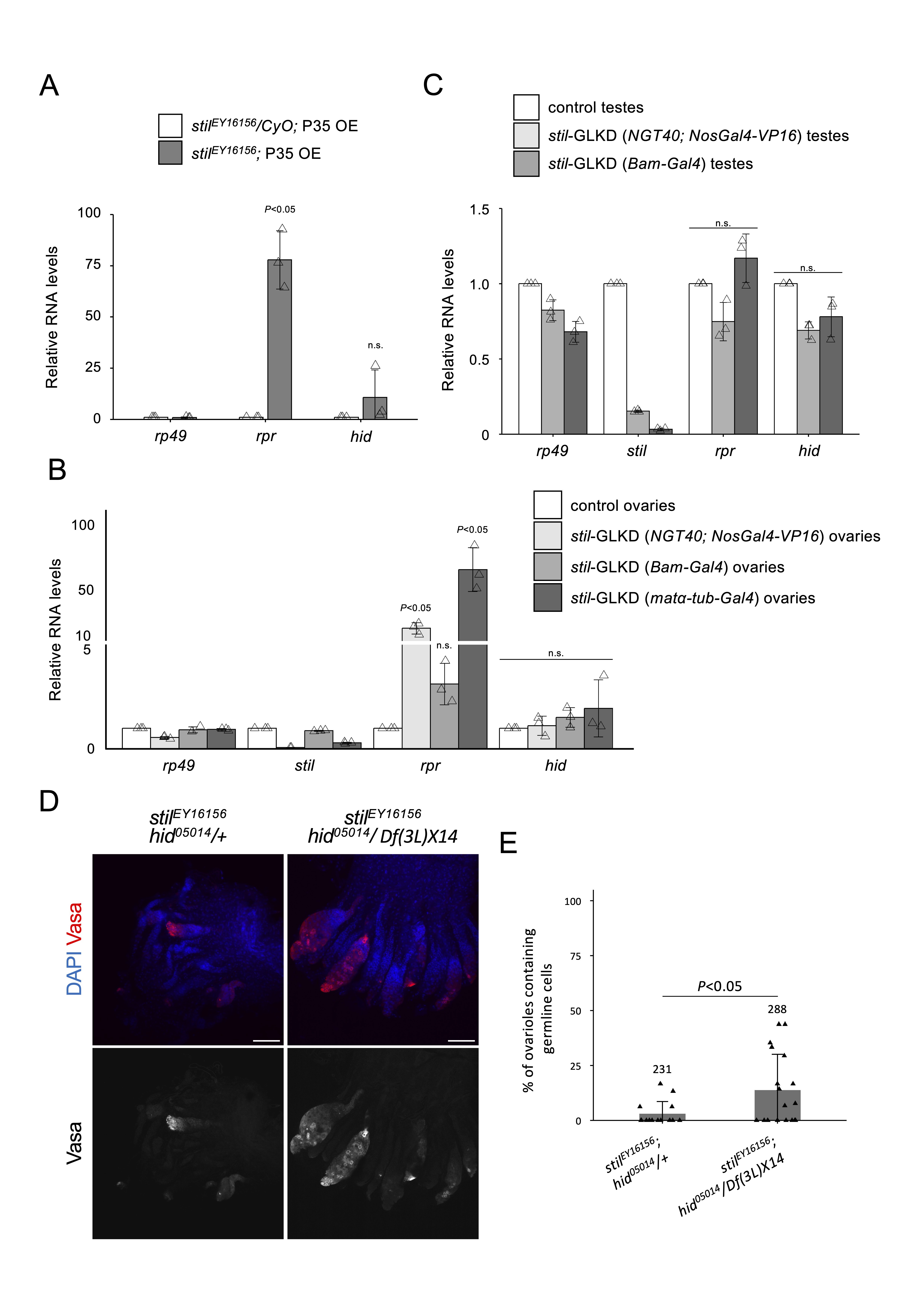

Supplement: S5 Fig — (A-C) Quantitative RT-PCR are performed on ovaries from germline-rescued stil mutants, as well as ovaries and testes with germline-specific stil knockdown using distinct drivers. Expression levels are normalized to αtub with rp49 serving as an internal control. (A) Early-stage ovaries are isolated by dissecting stage 4–5 oocytes from stilEY16156/CyO; P35 OE and stilEY16156; P35 OE flies (B) stil knockdown in female germline cells is achieved using NGT40-Gal4; NosGal4-VP16, Bam-Gal4, and matα-tub-Gal4 drivers. (C) stil knockdown in male germline cells is achieved using NGT40-Gal4; NosGal4-VP16 and Bam-Gal4 drivers. Error bars represent standard deviation (s.d.). (D) Immunostaining of stilEY16156; hid05014/+ and stilEY16156; hid05014/Df(3L)X14 ovaries with antibody against Vasa (red) with DAPI (blue). Scale bar: 50 μm. (E) Quantification of the percentage of ovarioles containing germline cells per ovary in 2–3-day-old females. Each dot represents an individual ovary. Genotypes are indicated below the graph and the number of germarium assessed is noted above each bar. Error bars represent standard deviation (s.d.). (JPG) [file pgen.1012041.s005.jpg]

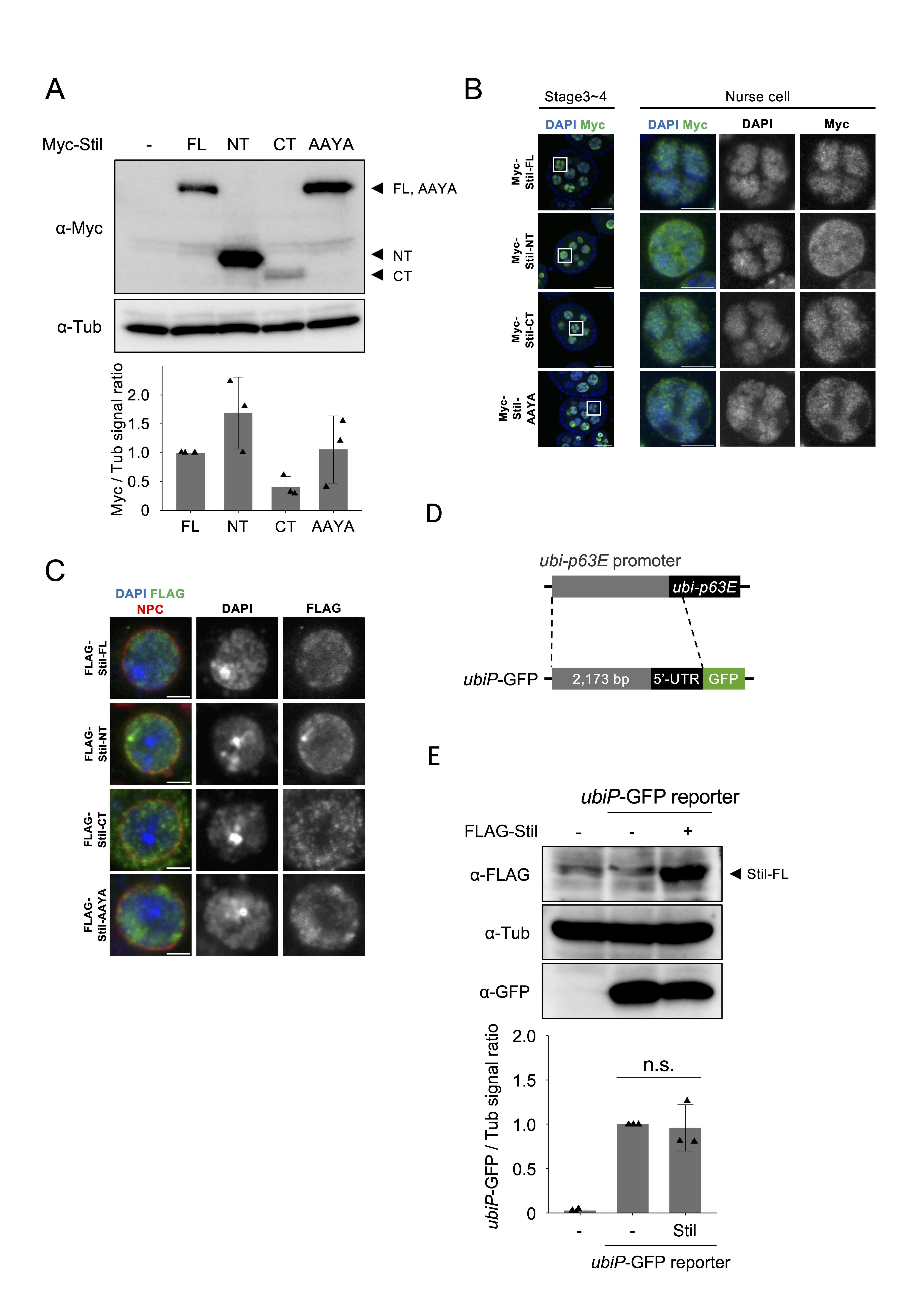

Supplement: S6 Fig — (A) Western blot analysis of 6×Myc-tagged Stil variants (FL, NT, CT, and AAYA) driven by NGT40-Gal4; NosGal4-VP16, with y w as a control. Stil variants were detected with anti-Myc, and α-Tubulin (αTub) served as a loading control. Arrowheads indicate Stil variant proteins. The lower panel shows quantification of the Myc/αTub signal ratio normalized to FL. Error bars indicate standard deviation (s.d.) (n = 3). (B) Immunofluorescence images showing Myc-tagged Stil variants (green) and DNA (DAPI, blue) in stage 3~4 egg chambers of the ovaries. Insets show enlarged views of nurse cell nuclei. Scale bar: 20 μm (egg chamber) and 5 μm (nurse cell nuclei). (C) Immunostaining of S2 cells expressing FLAG-tagged Stil variants with an anti-FLAG (green) antibody and DAPI (blue). Scale bar: 2 μm (D) Schematic representation of the ubiP-GFP reporter construct, in which the 2,173 bp upstream region and 5’-UTR of the ubi-p63E gene is fused to GFP. (E) Reporter assay in S2 cells followed by western blot analysis to detect GFP expression. Co-transfection of the ubiP-GFP reporter and FLAG-tagged Stil-FL were performed in S2 cells. An arrowhead indicates Stil-FL protein. α-Tubulin (αTub) is used as a loading control. The lower panel quantifies the GFP/αTub signal ratio, normalized to that in S2 cells transfected with the reporter alone. Error bars indicate standard deviation (s.d.) (n = 3). (JPG) [file pgen.1012041.s006.jpg]

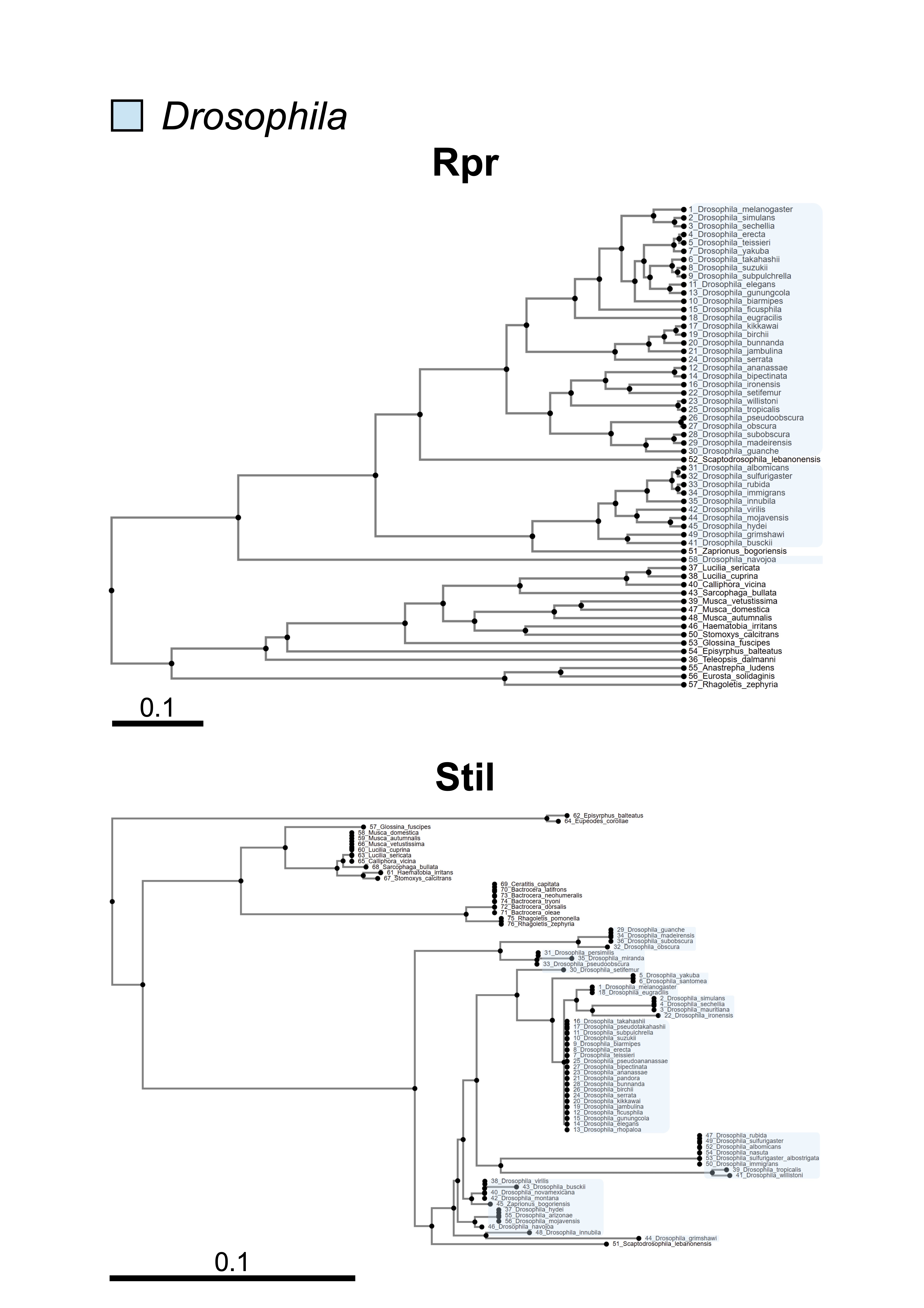

Supplement: S7 Fig — Homologs of Drosophila melanogaster Rpr and Stil were identified by BLASTp, aligned, and analyzed phylogenetically. Homologs are present across Dipteran lineages, with the genus Drosophila highlighted in blue. Branch lengths indicate the expected number of substitutions per site, as shown by the scale bar. (JPG) [file pgen.1012041.s007.jpg]
