# Supplementary material for: Transcriptional repression of reaper by Stand still ensures female germline development in Drosophila
Source: PLoS Genet. 2026 Mar 5;22(3):e1012041. doi: 10.1371/journal.pgen.1012041 (PMC12962474; doi:10.1371/journal.pgen.1012041)
Supplement: Attachment — Submitted filename: 158625_1_rebuttal_3554444_t4czd0.pdf [file pgen.1012041.s014.pdf]

Full Revision

Review COMMONS

Manuscript number: RC-2025-03058
Corresponding author(s): Toshie, KAI

[Please use this template only if the submitted manuscript should be considered by the affiliate journal as a full revision in response to the points raised by the reviewers.

If you wish to submit a preliminary revision with a revision plan, please use our "Revision Plan" template. ***It is important to use the appropriate template to clearly inform the editors of your intentions.****]*

1. General Statements [optional]

This section is optional. Insert here any general statements you wish to make about the goal of the study or about the reviews.

We sincerely thank the reviewers for their thorough evaluation of our work and for providing constructive and insightful comments. We have carefully addressed all the points raised and revised the manuscript accordingly, while also incorporating additional minor changes as detailed below. We believe that these revisions have further strengthened the clarity, rigor, and overall presentation of our study.
To our knowledge, this is the first report to identify *stand still* (*stil*) as a female germline–specific transcriptional repressor of the pro-apoptotic gene *reaper* (*rpr*). This is significant because the mechanisms regulating apoptosis in germline cells remain poorly understood, despite their essential role in preserving reproductive capacity. First, we present direct experimental evidence that Stil binds to the upstream regulatory region of *rpr* and represses its transcription via its BED-type zinc finger domain. We believe this provides an important step toward understanding how the core apoptotic inducer *rpr* is regulated in a cell-type–specific manner. Second, we show that in the absence of *stil*, differentiated germline cells undergo apoptosis, whereas undifferentiated germline stem cell-like cells remain resistant. This resistance is associated with a closed, H3K9me3-enriched chromatin state at the *rpr* locus, suggesting that chromatin-based silencing cooperates with Stil repression. Together, these findings reveal dual mechanisms: Stil-mediated transcriptional repression and chromatin-mediated silencing, that safeguard the female germline against inappropriate *rpr*-induced apoptosis. Third, our evolutionary analyses show that *stil* and *rpr* are restricted to Diptera, while the BED-type zinc finger domain of Stil and the IAP-binding motif of Rpr are broadly conserved across metazoans. These results provide new insights into the evolutionary relationships between lineage-specific regulators and conserved apoptotic pathways.

2. Point-by-point description of the revisions

This section is mandatory. Please insert a point-by-point reply describing the revisions that were already carried out and included in the transferred manuscript.

Reviewer #1 (Evidence, reproducibility and clarity (Required)):

Summary: This work by Matsui et al. examined the function of a gene Stand Stil (stil) in Drosophila in regulation of germ cell death in the female germline. They show that stil mutants contain many apoptotic cells, leading to germ cell loss and infertility. Gene expression analysis showed upregulation of pro-apoptotic genes such as rpr in stil mutant. DamID experiment further showed that stil binds to rpr promoter region to repress its expression. Additionally, they also show that undifferentiated germ cells are resistant to cell death in stil mutant (but stil mutant still eventually loses all germ cells).

Major comments: Overall, experiments adhere to a general standard of rigor, and each result is fairly convincing. In that sense, this paper warrants publication, as a paper that revealed a new gene important for preventing germ cell death. With that said, I feel that this paper does not reveal a new biological insight. In a nutshell, this paper is about a transcriptional repressor for pro-apoptotic gene, hence its depletion leads to cell death. Data is solid and the conclusion is well supported. But the readers will be left wondering why nature implemented such control? Unless one can show what kind of defects stil rpr double mutant (which rescues germ cell loss phenotype) exhibits, there is no insight why the balance of pro-apoptotic gene and its repressor is important. The paper discusses the 'molecular' mechanisms that explain the phenomenon, but it does not provide insights. The lack of conceptual advancement is the limitation of this work.

Response:
We thank the reviewer for pointing out a biological insight into the evolutionary rationale underlying the adoption of such a regulatory mechanism in nature. To address this point, we assessed the evolutionary conservation of *rpr* and *stil* through BLAST searches and comparative analyses. Our results showed that both genes are Diptera-restricted, whereas their key domains (the *rpr* IAP-binding motif and the Stil BED finger) are widely conserved across metazoans. In this phylogenetic context, we propose that Stil acts as a dedicated repressor of *rpr* in the *Drosophila* female germline, thereby establishing an apoptotic control architecture in which *hid* predominates and *rpr* is repressed by Stil. This explains why the balance between a potent effector (Rpr) and its repressor (Stil) is critical in oogenesis; preventing catastrophic germline loss while preserving *hid*-mediated responsiveness.

We have incorporated these phylogenetic analyses and the perspective into the revised Discussion section as follows.

Revised Page 22, Line 475; *rpr* is conserved only within Diptera, although its IAP-binding motif, essential for apoptosis induction, is broadly conserved across metazoans (Du et al., 2000; Gottfried et al., 2004; Hegde et al., 2002; Shi, 2002; Verhagen et al., 2000; Vucic et al., 1998; Wing et al., 2001; L. Zhou, 2005) (Fig. S7). Similarly, *stil* is also restricted to Diptera, predominantly within Drosophila, whereas its BED-type zinc finger domain is widely conserved among diverse organisms (Aravind, 2000; Hayward et al., 2013; Tue et al., 2017b; H. Zhou et al., 2016). Phylogenetic patterns across Diptera are consistent with a model in which *stil* acts as a dedicated repressor of *rpr* in the *Drosophila* germline cells (Fig. S7). Due to its potent pro-apoptotic activity, *rpr* must be stringently repressed in a spatiotemporal manner through mechanisms that are specific to both cell type and developmental stage. During embryogenesis, repression of *rpr* is mediated by the Dpp-signaling factor Shn, which binds to the *rpr* regulatory region, whereas in intestinal stem cells (ISCs), its expression is suppressed through chromatin conformation. In *Drosophila* female germline cells, *hid* serves as the primary regulator of apoptosis, while *rpr* activity is generally suppressed (Park et al., 2019; Xing et al., 2015). However, *rpr* mutants exhibit reduced fertility despite producing viable eggs (Fig. 3H), suggesting that *rpr*-mediated apoptosis may be required for proper egg development. Accordingly, we propose that *stil* restrains *rpr* in the *Drosophila* female germline, allowing *hid* to predominate in apoptotic regulation.

Full Revision

New Fig. S7;

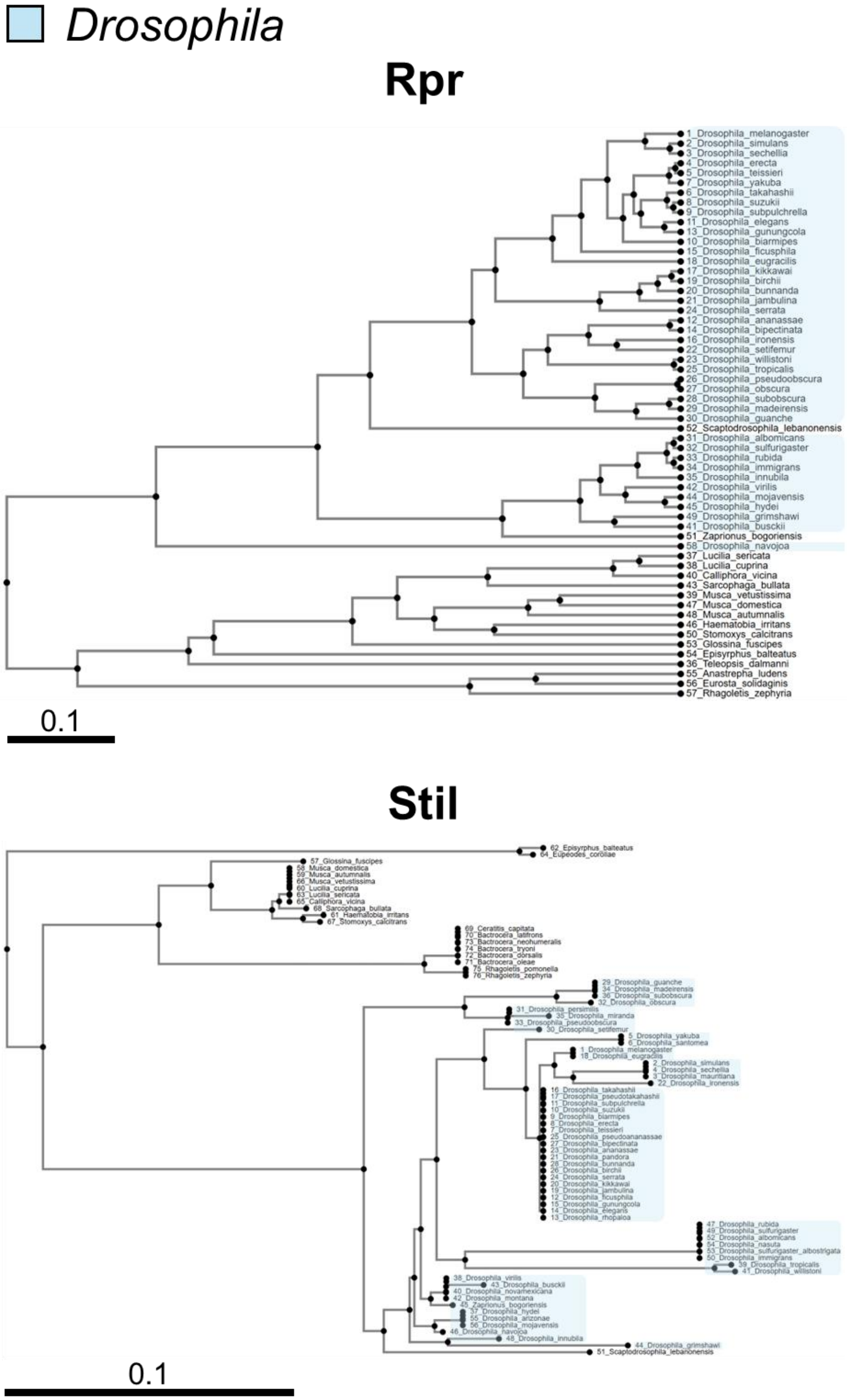

The legend of new Fig. S7;

Figure S7 Conservation of Rpr and Stil within Diptera

Homologs of *Drosophila melanogaster* Rpr and Stil were identified by BLASTp, aligned, and analyzed phylogenetically. Homologs are present across Dipteran lineages, with the genus *Drosophila* highlighted in blue. Branch lengths indicate the expected number of substitutions per site, as shown by the scale bar.

Minor comments: Although this is a minor point, and this is not specifically pointing a finger at the author of this paper, I really don't like the term 'safeguard'. This term is now overutilized to add hype to papers, when 'is necessary' is sufficient. In this case, unless the answer is provided as to 'against what stil is safeguarding germ cells', this term is not meaningful. For example, if one can show that stil specifically senses germline-specific threat and tweaks the regular apoptotic pathway based on germline-specific needs, then the term 'safeguard' may be warranted.

Response:
In light of the reviewer's comment, we have revised the title of the manuscript to replace 'safeguard' with 'ensure,' which better reflects the demonstrated function of Stil without overstating its role. The new title of the manuscript is: 'Transcriptional Repression of *reaper* by Stand Still Ensures Female Germline Development in *Drosophila*'

Reviewer #2 (Evidence, reproducibility and clarity (Required)):

In this well-executed study, Matsui et al. investigate how the female Drosophila germline prevents inappropriate apoptosis during development. They identify stand still (stil) as a key germline-specific repressor of apoptosis. Stil mutant flies are homozygous viable but female sterile due to widespread germ cell loss at the time of eclosion, which is driven by activation of the pro-apoptotic gene reaper (rpr) and caspase-dependent cell death. Germline-specific expression of anti-apoptotic factors such as p35 can rescue this phenotype, confirming that the defect lies in apoptotic regulation. The authors show that Stil directly represses rpr transcription through its BED-type zinc finger domain. Notably, undifferentiated germline cells remain resistant to apoptosis in the absence of stil, which the authors attribute to a silenced chromatin state at the rpr locus, marked by H3K9me3. These findings support a dual mechanism of protection: transcriptional repression of rpr by Stil, and a potential parallel chromatin-based silencing mechanism operating specifically in undifferentiated cells.
Major Issues:
1. Clarify cell identity in Figure 2E: It is unclear whether the apoptotic cells shown are somatic or germline in origin. Including a somatic marker such as 1B1 would allow the reader to clearly distinguish the apoptotic population and better interpret the figure.

Response:

We thank the reviewer for this helpful suggestion. Occasionally, the signal of the germline marker Vasa can be attenuated in dying germline cells. As suggested by the reviewer, we also tested α-Spectrin (a plasma membrane and fusome marker) instead of 1B1 together with TUNEL labeling, but this approach did not clearly distinguish somatic from germline apoptotic cells. To directly clarify cell identity, we now provide an improved co-stained image in which TUNEL-positive nuclei are surrounded by Vasa-positive cytoplasm, indicating a germline origin. Figure 2E has been updated accordingly.

New Fig. 2E;

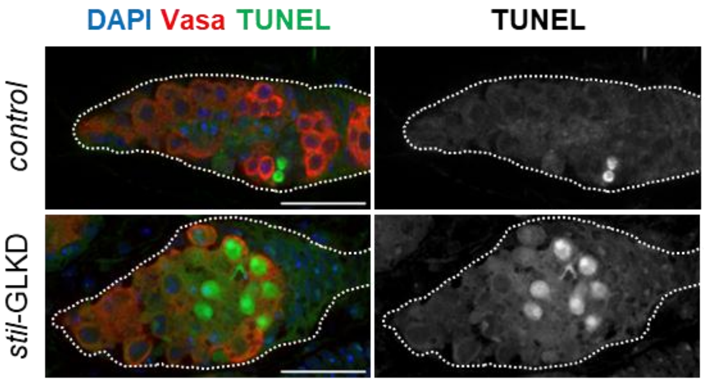

2. Quantification of undifferentiated cells in mutants: There appears to be inconsistency in the representation of undifferentiated germ cells across figures. Early panels show near-complete germline loss, while later analyses focus on undifferentiated cells that are reportedly apoptosis-resistant. The authors should quantify the proportion of ovarioles retaining undifferentiated cells and present this data in Figure 1 or the supplements to resolve this discrepancy.

Response:
Thank you for raising the important point regarding the apparent inconsistency in the representation of undifferentiated germ cell populations. In early panes (Fig.1C, D), we analyzed adult ovaries of *stil* loss-of function mutants where all germline cells including undifferentiated germline stem cells (GSCs) are almost completely lost (Fig. 1C), showing nearly 100% agametic ovarioles. However, in later analysis such as those in Fig. 5A, B, we showed 3rd instar-larval ovaries of *stil* loss-of function mutants containing a few surviving germline cells nearby the future cap cell, the niche providing stem cell ligand, Decapentaplegic (Dpp) (Xie & Spradling, 1998). This suggests that Dpp-responsive undifferentiated germline cells may be relatively resistant to apoptosis caused by *stil* loss.
Indeed, the GSC-like cells generated by the overexpression of a constitutively active form of Dpp receptor, Thickveins (Tkv.CA) or loss of the differentiation factor *bam*, were resistant to apoptosis caused by *stil* loss (Fig. 5C, D). These GSC-like cells may possess enhanced stemness, owing to either excessively elevated Dpp signaling or complete loss of *bam*, which could lead to stronger repression of *rpr* expression through tighter chromatin compaction.

We added this argument in the Results section of the revised manuscript as follows.

> Revised Page 16, Line 361; Compared to GSCs, which were almost completely lost in *stil* mutants, GSC-like cells may retain a more robust stemness owing to the extremely elevated Dpp signaling pathway, potentially resulting in stronger repression of *rpr* expression.

3. Interpretation of chromatin state at the rpr locus: The claim that H3K9me3, but not H3K27me3, marks the rpr locus is not fully convincing given the low ChIP-seq signal shown. Including a comparison to a known positive control locus would strengthen the argument. Alternatively, the authors could broaden the discussion to include global chromatin reorganization during germ cell to maternal transition, as reported in Kotb et al., 2024 and how such changes may impact rpr accessibility. Also stl mutant rescued with P53 have a "string of pearls" phenotype that are associated with germ cell to maternal transition defects (Figure S3, p53 OE)

Response:
We thank the reviewer for the thoughtful and constructive comment regarding the interpretation of chromatin state at the *rpr* locus. To strengthen the inference that the *rpr* locus shows H3K9me3 enrichment, whereas clear H3K27me3 enrichment is not evident, we have now included ChIP-seq signal profiles for known positive control loci, using *light* (*lt*) as an H3K9me3-enriched locus (Akkouche et al., 2017; Greil et al., 2003) and *Ultrabithorax* (*Ubx*) as a canonical H3K27me3 target (Torres-Campana et al., 2022). These comparisons support our interpretation that H3K9me3, rather than H3K27me3, characterize chromatin around the *rpr* locus in GSCs. Accordingly, while we do not exclude a minor H3K27me3 contribution, our analyses indicate H3K9me3 as the predominant signature at *rpr* in GSCs.

New Fig.6B and 6C;

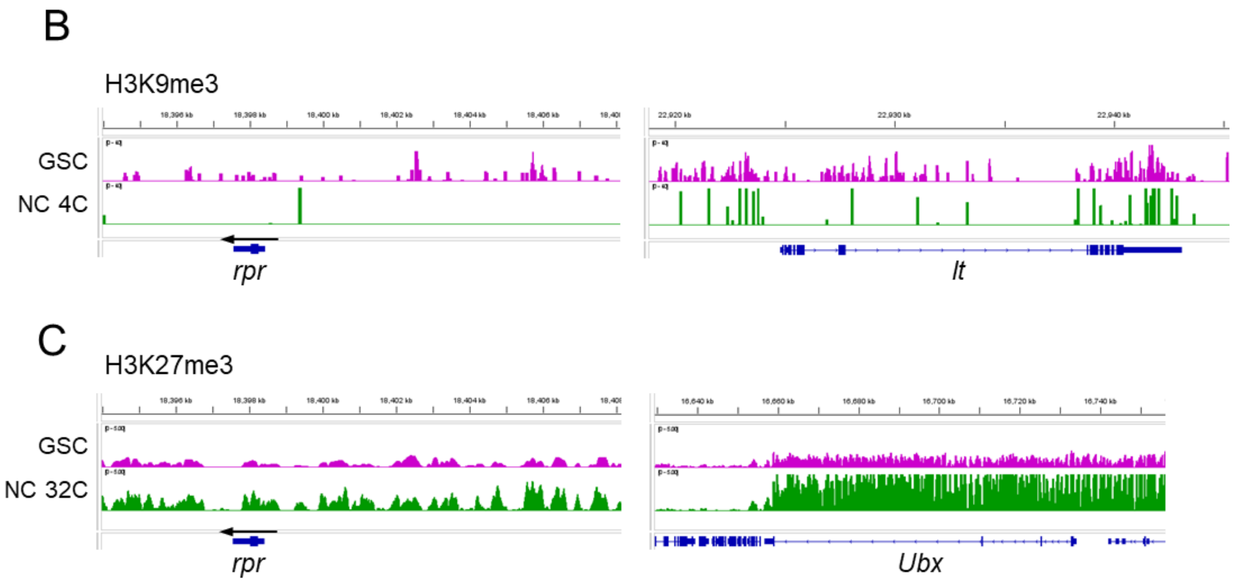

The legend of new Fig. 6B and Fig. 6C;

(B) H3K9me3 ChIP-seq signal at the *rpr* locus and the *lt* locus (H3K9me3-positive control) in GSCs and 4C NCs. (C) H3K27me3 ChIP-seq signal at the *rpr* locus and the *Ubx* locus (H3K27me3-positive control) in GSCs and 32C NCs.

A sentence of Result section was revised as below.

Revised Page 17, Line 396; As internal controls, we confirmed H3K9me3 enrichment at the *light* (*lt*) locus and H3K27me3 enrichment at the *Ultrabithorax* (*Ubx*) locus, consistent with their established chromatin states (Akkouche et al., 2017; Greil et al., 2003; Torres-Campana et al., 2022); relative to these controls, the *rpr* locus shows H3K9me3 but no clear H3K27me3 enrichment in GSCs.

Regarding the suggestion to broaden the discussion to include global chromatin reorganization during the germline-to-maternal transition, as reported in Kotb et al., 2024, we agree that this is an important avenue for understanding *rpr* accessibility. The “string of pearls” phenotype observed in *stil* mutants rescued with P35 overexpression (Figure S3) is consistent with perturbations during this transition. However, a detailed analysis of such chromatin reorganization and its potential impact on *rpr* regulation lies beyond the scope of the present study and represents a valuable direction for future work.

4. Broader analysis of rpr regulation in somatic cells: It would be informative to examine publicly available chromatin or transcriptional data for the rpr locus in somatic ovarian cells. This could

help clarify whether rpr regulation by Stil is truly germline-specific or reflects broader developmental trends. This will also clarify why the flies are homozygous viable but female sterile.

Response:
We thank the reviewer for this insightful suggestion. We agree that exploring chromatin accessibility and transcriptional regulation at the *rpr* locus in somatic ovarian cells would provide valuable insights into tissue- or cell-type-specific chromatin environments that influence *rpr* expression.
However, to our knowledge, there are currently no publicly available ATAC-seq or comparable chromatin datasets for purified ovarian somatic cells, including follicle cells or ovarian somatic cells (OSCs). As such, we are unable to incorporate this analysis in the current study. Nevertheless, we fully recognize the importance of this line of inquiry and consider it a valuable direction for future research.

Reviewer #3 (Evidence, reproducibility and clarity (Required)):

Summary:
This manuscript describes the characterization of stand still (stil), a previously identified gene needed for germ cell survival in Drosophila. The molecular function of Stil has until now remained poorly understood. This new work shows that loss of stil results in reaper (rpr)-dependent apoptosis within female germ cells. Loss of rpr suppresses many of the phenotypes observed in stil mutants. Experiments performed using Drosophila cell culture suggest that Stil binds to elements within the rpr promoter. DamID and structure/function experiments indicate that Stil likely directly represses the transcription of rpr within germ cells.

In general, the experiments are well executed, and the data largely support the basic claims of the authors. Replicates are included and appropriate statistical analyses have been provided. The text and figures clear and accurate. Appropriate references were cited. There are a few things the authors should address or rephrase before publication.

On page 9 line 190-192. The authors state "Altogether, these findings indicate that the loss of stil function not only triggers apoptosis that can be suppressed by apoptosis inhibitors but also causes defects in oogenesis progression that are not rescued by blocking cell death." Failure to rescue defects during mid-oogenesis could be due to insufficient transgene expression. Indeed, loss of rpr appears to rescue the fertility of stil mutants. The conclusions of this section should be restated.

Response:
We agree that the failure to rescue mid-oogenesis defects by P35 overexpression may, at least in part, be due to insufficient transgene expression. This explanation is particularly plausible

given that loss of *rpr* more effectively restored fertility in *stil* mutants. As suggested by the reviewer, we have revised the relevant sentences, to avoid misinterpretation as below.

Revised Page 9, Line 191; Altogether, these findings indicate that the loss of *stil* function triggers apoptosis that can be suppressed by apoptosis inhibitors.

Revised Page 12, Line 253; The complete rescue of germline survival in *stil rpr* double mutants also suggests that the failure of P35 overexpression to restore mid-oogenesis defects may partly reflect insufficient transgene expression (Fig. S3).

The authors should present the overlap between genes that change expression in a stil mutant and those in which the DamID experiments indicate are directly bound by Stil protein. DamID can sometimes give spurious results depending on expression levels. Further discussion along this point is necessary.

Response:
We thank the reviewer for raising this issue. As suggested, we have now analyzed the overlap between genes that are differentially expressed in *stil* mutant ovaries (identified by RNA-seq with *stil* mutant expressing P35) and genes that are potentially bound by Stil based on DamID-seq data (promoter-proximal peaks ≤1 kb) as Supplementary Table 4. The list includes genes with DamID peaks within promoter regions and that also exhibit significant differential expression ($|\log2FC| > 1$, adjusted $p < 0.01$) in the RNA-seq dataset.
The overlap between DamID-seq and RNA-seq comprises 682 genes, including *rpr*, supporting the idea that Stil regulates *rpr* expression through interaction with its upstream promoter region. However, the detected peak signal at *rpr* was 3.41, which was not that strong, suggesting that Stil may also bind to and regulate other genes in female germline cells. Investigating the potential role of Stil in regulating other genes represents an important future direction of our study.

We have included this analysis and argument in the revised manuscript as below.

Revised Page 13, Line 280; A total of 682 genes with Stil-enriched peaks detected at promoter regions (≤1 kb) showed significantly altered expression in RNA-seq analysis of *stil* mutants expressing P35, including *rpr* (Supplementary Table 4).

Revised Page 20, Line 440; Notably, the DamID peak intensity at the *rpr* locus reached 3.41, which is moderate rather than strong (Supplementary Table 4). This suggests that, in addition to repressing *rpr*, Stil may bind to and regulate other genomic loci in the female germline. Investigating the repertoire of Stil target genes and elucidating their roles in germline cells will be an important future direction of this study.

For structure function experiments, a western blot showing expression levels of the different transgenes in ovaries should be included.

Response:
We thank the reviewer for this helpful comment. To address this point, we examined the expression levels of the four Stil variants (FL, NT, CT, and AAYA) in ovaries driven by a germline driver under a wild-type background using Western blotting. The representative blot and quantification from three biological replicates showed comparable expression levels among the variants, with the CT variant displaying a slightly reduced signal. Importantly, AAYA showed expression comparable to FL yet, like CT, failed to rescue, indicating that the rescue failure is not explained by expression-level differences. These data instead support a requirement for the BED-type zinc finger for Stil function in the germline. While we cannot fully exclude a minor contribution from the slightly lower expression of the CT variant to the lack of rescue, the AAYA result argues that loss of BED-type zinc-finger function is the primary cause; we note this caveat in the revised text. The corresponding data are now presented in Figure S6A of the revised manuscript.

New Fig. S6A;

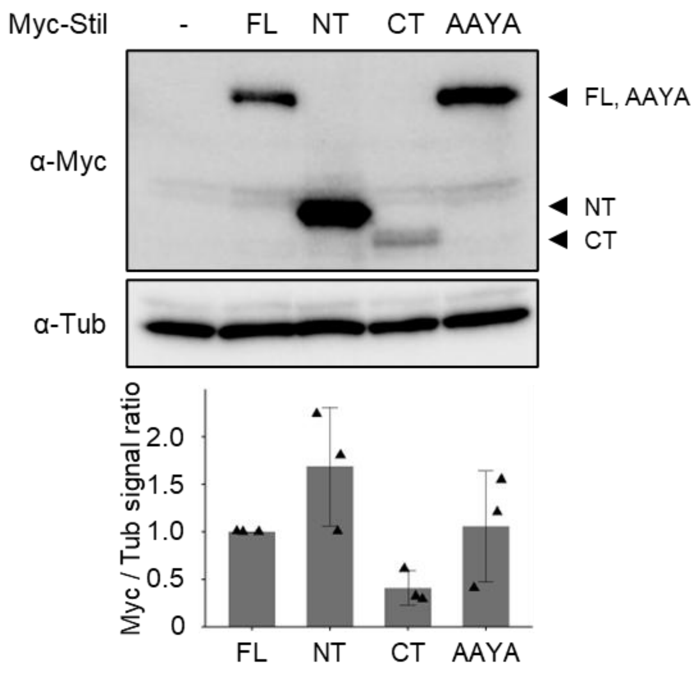

The legend of new Fig. S6A;

(A) Western blot analysis of 6×Myc-tagged Stil variants (FL, NT, CT, and AAYA) driven by *NGT40-Gal4; NosGal4-VP16*, with *y w* as a control. Stil variants were detected with anti-Myc, and α-Tubulin (αTub) served as a loading control. Arrowheads indicate Stil variant proteins.

The lower panel shows quantification of the Myc/αTub signal ratio normalized to FL. Error bars indicate standard deviation (s.d.) (n = 3).

A sentence of Result section was revised as below.

Revised Page 13, Line 291; The expression of all four Stil variant proteins from the transgenes was confirmed, although Stil-CT showed a slightly reduced expression level (Fig. S6A)

Revised Page 14, Line 305; Although CT shows slightly lower expression, AAYA fails to rescue despite FL-like expression, indicating that expression level is not limiting and that loss of the BED-type zinc finger underlies the phenotype.

"With the addition of the new Fig. S6A, the following figure labels have been updated;

Fig. S6A →S6B
Fig. S6B → S6C
Fig. S6C → S6D
Fig. S6D → S6E

Individual data points should be shown in each graph in place of simple bar graphs. This type of presentation was inconsistent throughout the paper.

Response:
We thank the reviewer for this constructive comment. In line with the reviewer's suggestion, we have revised the relevant graphs to include individual data points overlaid on bar plots with error bars. This modification enables readers to better assess data variability. We also ensured consistency in data presentation among the revised figures while maintaining clarity throughout the manuscript.

Reference "G & D., 1997" should be properly formatted.
Page 6 line 117 and 121- a couple of instances where "cell" should be "cells"
Page 14 line 304- typo "Still"

Response:
As suggested, we have revised all figures to display individual data points in each graph instead of using simple bar graphs. This change has been applied consistently throughout the manuscript to improve data transparency and readability. The revised figures include Figure 1A, 2B, S1A, and S2A.

We have also corrected the following textual issues;

・The reference “G & D., 1997” has been properly formatted as “Pennetta & Pauli, 1997”.
・On page 6, lines 119 and 123, “cell” has been corrected to “cells” to ensure grammatical accuracy.
・On page 14, line 315, the typo “Still” has been corrected to “Stil”.

Reviewer #3 (Significance (Required)):

The significance of the work lies in characterizing a previously unknown function of Stil. By showing that Stil acts to repress transcription of the cell death gene *rpr*, the authors provide new insights into how programmed cell death is regulated in the Drosophila female germline. Readers interested in reproductive biology, cell death, chromatin, and general developmental biology will find value in these new findings.

One thing to consider is the possibility that Stil represses *rpr* in the context of the double strand breaks that form during meiosis. Experiments in the paper indicate that *stil* knockdown results in TUNEL labeling in region 2A/2B of the germarium. The authors should consider co-labeling for a meiosis marker (C(3)G or gammaH2Av) to see if this PCD correlates with this expression. In addition, they could test whether loss of Spo11 (mei-W68) suppresses *stil* phenotypes during early germ cell development. Relating the function of Stil to repression of cell death during this critical time of germ cell development would elevate the impact and significance of the paper. However, this may be considered beyond the scope of the current study.

Response:
We deeply thank the reviewer for this insightful and thought-provoking suggestion.
As suggested, we conducted co-staining with γH2Av (DBS marker), as well as genetic interaction experiments with *Spo11* (*mei-W68*) mutants to address this question shown below. In region 2 across all genotypes including *y w* control, and *stil* heterozygous and homozygous ovaries expressing P35, γH2Av signals were discernible and subsequently lost in region 3 through the meiotic recombination-specific DNA repair program (Additional Figure A). In *stil* mutants, however, an additional strong γH2Av signal was specifically observed in the oocyte, beyond the expected meiotic pattern.
Furthermore, loss of meiotic recombination factors, including *mei-W68*, in *stil* mutants partially rescued the germline loss phenotype, although not to the same extent as in *rpr* mutants (Additional Figure B, C: 43.5 % in *mei-W68*-GLKD, 23.9 % in *mei-P22*^{P22} and 12.8 % in *vilya*^{826} versus 100 % with loss of *rpr* in Fig. 3E, F of the revised manuscript). These findings suggest that accumulation of meiotic DSBs is not the main cause of *rpr* upregulation in *stil* mutants. We feel that these analyses are beyond the scope of the current study, which focuses on identifying Stil as a transcriptional repressor of *rpr* and characterizing its role in germline apoptosis. Elucidating other mechanisms that elevate *rpr* expression in *stil* mutants will be the focus of future work. Hence, we are providing these data here for the reviewer’s reference, but if the reviewer prefers, we would be happy to incorporate them into the manuscript.

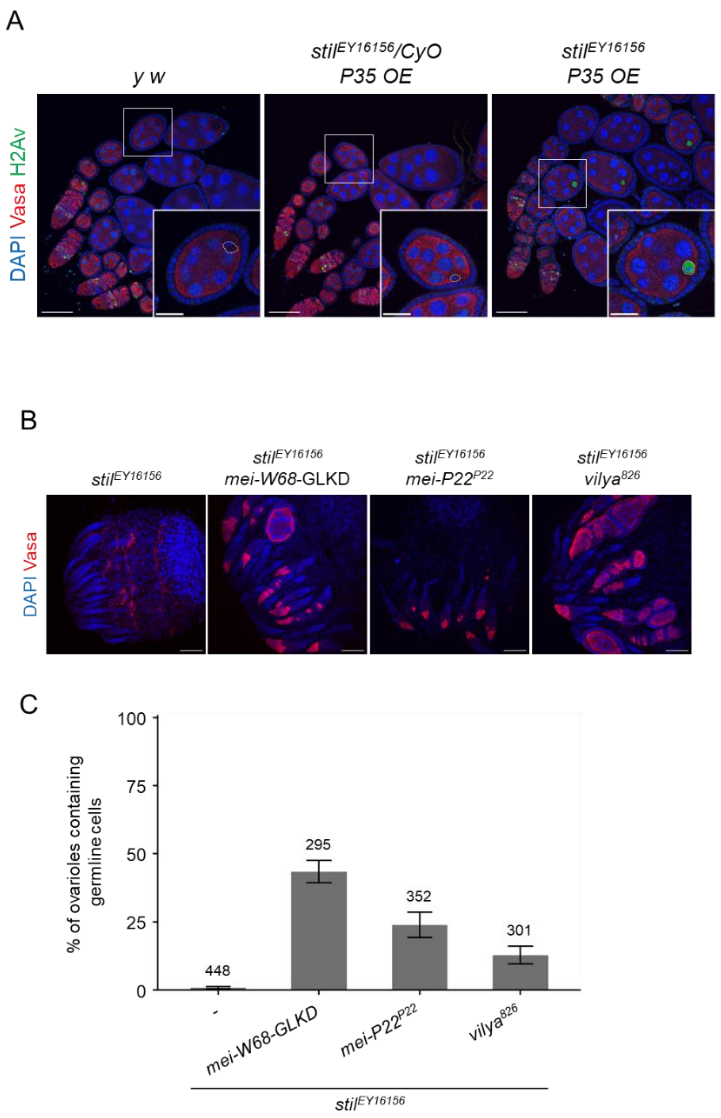

Additional Figure (A) Immunostaining of ovarioles from *y w*, *stil^{EY16156}/CyO; P35 OE (NGT40; NosGal4-VP16> P35)*, *stil^{EY16156}; P35 OE* flies with antibody against DNA double-strand break marker H2Av (green), Vasa (red), and DAPI (blue). Insets show enlarged views of egg chamber. White dots indicate oocyte nuclei, Scale bar: 50 µm (ovariole) and 20 µm (egg chamber). (B) Immunofluorescence of Vasa (red) and DAPI (blue) in ovaries from *stil^{EY16156}*, *stil^{EY16156}; mei-W68*-GLKD (driven by *NGT40; NosGal4-VP16*), *stil^{EY16156}; meiP22^{P22}*, and *stil^{EY16156}; vilya^{826}*. Scale bar: 50 µm. (C) Quantification of the percentage of ovarioles containing germline cells in 2–3-day-old females. The genotypes of females are indicated below the x-axis, and the number of germaria analyzed is shown above each bar. Error bars represent the standard deviation (s.d.).

Akkouche, A., Mugat, B., Barckmann, B., Varela-Chavez, C., Li, B., Raffel, R., Pélisson, A. & Chambeyron, S. (2017). Piwi Is Required during Drosophila Embryogenesis to License Dual-Strand piRNA Clusters for Transposon Repression in Adult Ovaries. *Molecular Cell*, *66*(3), 411-419.e4. https://doi.org/10.1016/j.molcel.2017.03.017

Greil, F., Kraan, I. van der, Delrow, J., Smothers, J. F., Wit, E. de, Bussemaker, H. J., Driel, R. van, Henikoff, S. & Steensel, B. van. (2003). Distinct HP1 and Su(var)3-9 complexes bind to sets of developmentally coexpressed genes depending on chromosomal location. *Genes & Development*, *17*(22), 2825–2838. https://doi.org/10.1101/gad.281503

Röper, K. & Brown, N. H. (2004). A Spectraplakin Is Enriched on the Fusome and Organizes Microtubules during Oocyte Specification in Drosophila. *Current Biology*, *14*(2), 99–110. https://doi.org/10.1016/j.cub.2003.12.056

Torres-Campana, D., Horard, B., Denaud, S., Benoit, G., Loppin, B. & Orsi, G. A. (2022). Three classes of epigenomic regulators converge to hyperactivate the essential maternal gene deadhead within a heterochromatin mini-domain. *PLoS Genetics*, *18*(1), e1009615. https://doi.org/10.1371/journal.pgen.1009615

Xie, T. & Spradling, A. C. (1998). decapentaplegic Is Essential for the Maintenance and Division of Germline Stem Cells in the Drosophila Ovary. *Cell*, *94*(2), 251–260. https://doi.org/10.1016/s0092-8674(00)81424-5
